# Advances in mechanism and regulation of PANoptosis: Prospects in disease treatment

**DOI:** 10.3389/fimmu.2023.1120034

**Published:** 2023-02-09

**Authors:** Peng Zhu, Zhuo-Ran Ke, Jing-Xian Chen, Shi-Jin Li, Tian-Liang Ma, Xiao-Lei Fan

**Affiliations:** ^1^XiangYa School of Medicine, Central South University, Changsha, Hunan, China; ^2^School of Anesthesiology, Guizhou Medical University, Guiyang, Guizhou, China; ^3^Department of Orthopedics, Xiangya Hospital, Central South University, Changsha, Hunan, China; ^4^Department of Orthopedics, Honghui Hospital, Xi’an Jiaotong University, Xi’an, China

**Keywords:** cell death, infection, PANoptosis, PANoptosome, PCD

## Abstract

PANoptosis, a new research hotspot at the moment, is a cell death pattern in which pyroptosis, apoptosis, and necroptosis all occur in the same cell population. In essence, PANoptosis is a highly coordinated and dynamically balanced programmed inflammatory cell death pathway that combines the main features of pyroptosis, apoptosis, and necroptosis. Many variables, such as infection, injury, or self-defect, may be involved in the occurrence of PANoptosis, with the assembly and activation of the PANoptosome being the most critical. PANoptosis has been linked to the development of multiple systemic diseases in the human body, including infectious diseases, cancer, neurodegenerative diseases, and inflammatory diseases. Therefore, it is necessary to clarify the process of occurrence, the regulatory mechanism of PANoptosis, and its relation to diseases. In this paper, we summarized the differences and relations between PANoptosis and the three types of programmed cell death, and emphatically expounded molecular mechanism and regulatory patterns of PANoptosis, with the expectation of facilitating the application of PANoptosis regulation in disease treatment.

## 1 Introduction

The life or death of individual cells determines the health or disease of multicellular organisms. Programmed cell death (PCD), which are active mechanisms designed to aid the body in its development and survival (1), is essential for the body to eliminate risk factors. Pyroptosis, apoptosis, and necroptosis are the clearest PCD pathways, which provide protection for the body to resist internal and external risk factors (2, 3). Pyroptosis is mediated by inflammasomes and is characterized by the formation of caspase-1 (CASP1)-dependent pores at the plasma membrane, cellular lysis, and release of inflammatory content (4). Under physiological conditions, apoptosis is the main form of cell death and a caspases-dependent non-inflammatory process characterized by the formation of apoptosomes. In infection or some other situations, the immune system will increase apoptosis sensitivity through the action of cytokines and chemokines or other pathways (5). Necroptosis is related to a variety of cytokines, mainly mediated by receptor-interacting protein kinase 3 (RIPK3), and characterized by cell swelling and membrane disruption (3, 6). Recent studies have revealed the existence of a highly coordinated cell death process in microbial infections, namely PANoptosis, which is closely related to the above three PCD pathways and essential for body immunity and limiting a wide range of pathogens (7, 8).

Current research on PANoptosis is mainly based on the pathogen infection model. PANoptosis was originally defined as a form of inflammatory cell death regulated by PANoptosome with key features of pyroptosis, apoptosis, and necroptosis (9). A study in 2016 demonstrated that pyroptosis, apoptosis, and necroptosis co-occur in macrophages infected with the influenza A virus (IAV) (10). Since then, PANoptosis has been found in other pathogen infections, such as *Yersinia*, *Candida albicans* (*C. Albicans*), and *Aspergillus fumigatus* (*A. fumigatus*) (11, 12). According to the summary of several studies, the cases of PANoptosis almost all involve the assembly of a class of complex polyprotein complexes (PANoptosome), which is essential for initiating cell death and sensing PAMPs, DAMPs, or other risk factors. The components that make up PANoptosome vary depending on the triggers, but almost all contain Z-DNA-binding protein 1 (ZBP1), absent in melanoma 2 (AIM2), RIPK3, RIPK1, apoptosis-associated speck-like protein containing a caspase recruitment domain (ASC), Fas-associated protein with death domain (FADD), CASP8, as well as key components that perform pyroptosis, apoptosis, and necroptosis. Therefore, PANoptosome has become the focus and entry point of research and regulation of PANoptosis.

**Figure 1:**
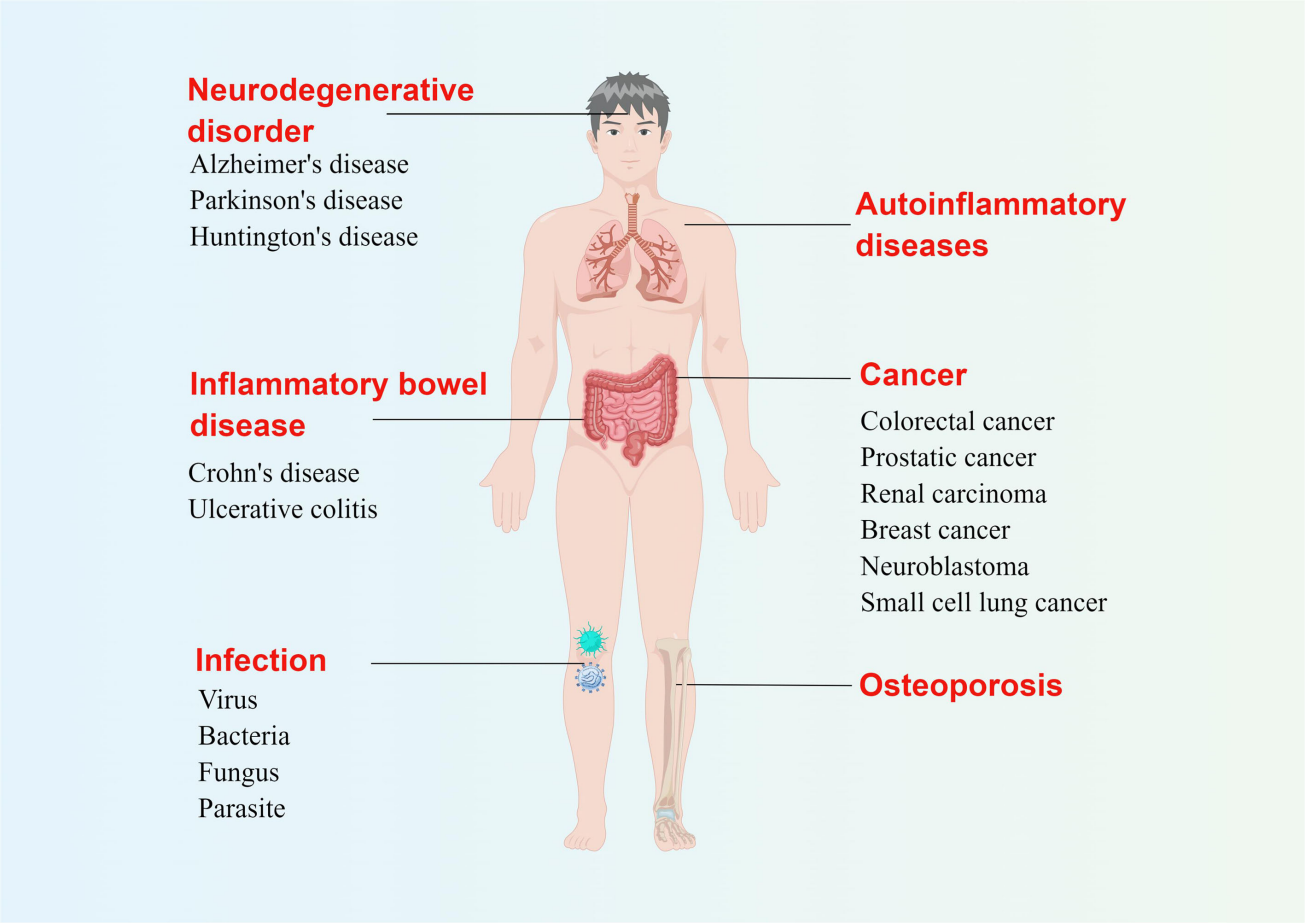
Possible diseases related to PANoptosis. Abnormal pyroptosis, apoptosis, or necroptosis can lead to a variety of diseases in the human body. The figure illustrates several diseases related to three kinds of programmed cell death at the same time, which are likely related to PANoptosis. By Figdraw (www.figdraw.com).

In addition to infection, PANoptosis may also be closely related to the occurrence and development of inflammatory diseases, neurodegenerative diseases, cancer, and other diseases (13, 14). Mutations in the *Pstpip2* gene cause autoinflammation through the NOD-like receptor family pyrin domain containing 3 (NLRP3)/CASP1- and CASP8-regulated PCD, which can be alleviated by simultaneously blocking pyroptosis, apoptosis, and necroptosis (15, 16). The role of pyroptosis in inflammatory bowel disease (IBD), has long been known. Overproduction of IL-1β dependent on the NLRP3 inflammasome is characteristic of Crohn’s disease (17), and disruption of the NLRP3 inflammasome also alleviates ulcerative colitis (18). In addition, inhibition of apoptosis can improve IBD (19), and the role of necroptosis of intestinal stem cells in the occurrence and development of IBD cannot be ignored (20). Considering the important role of pyroptosis, apoptosis, and necroptosis in IBD, we speculate that PANoptosis is closely related to the occurrence and development of this disease. Osteoporosis is associated with excessive cell death and loss. In the case of osteoporosis, the NLRP3 inflammasome not only promotes bone resorption but also destroys bone formation (21). Apoptosis of osteoblasts can cause osteoporosis (22), while inhibition of necroptosis is effective in reducing osteocyte loss (23). PANoptosis may also play an important role in neurodegenerative diseases such as Parkinson’s disease, Alzheimer’s disease, and Huntington’s disease. For example, mRNA for NLRP1, NLRP3, and CASP1/5/8 were up-regulate in Alzheimer’s disease (24), suggesting that PCD may contribute to its development. The relationship between PANoptosis and tumors is also a major research hotspot. Taking caspases as an example, CASP1 has a tumor suppressive effect in prostate cancer, kidney cancer, and colorectal cancer, while CASP3 is highly expressed in malignant breast cancer. The *caspase-8* gene is often silenced in malignant neuroendocrine tumors such as neuroblastoma, medulloblastoma, and small cell lung cancer (25). In general, PANoptosis disorders are involved in the occurrence and development of multiple systemic diseases in the human body (**Figure 1**).

It should not be ignored that the current research on PANoptosis is not mature, and the understanding of its trigger factors, regulation mechanism, and the types and assembly mechanism of PANoptosome still need to be further explored. This review mainly demonstrates the molecular mechanism of PANoptosis to promote the clinical treatment of multiple systemic diseases centered on PANoptosis regulation.

## 2 Pyroptosis, apoptosis, necroptosis, and PANoptosis

### 2.1 Pyroptosis

Pyroptosis is a type of programmed, lytic, and inflammatory cell death pathway that occurs following the assembly and activation of inflammasomes. As an innate immune mechanism, pyroptosis can enhance the host’s ability to resist pathogens (4). Inflammasomes are multimeric protein complexes, which usually consist of sensors, adaptor proteins, and the caspase family (26). The sensor is a pattern recognition receptor (PRR), such as nucleotide-binding domain-like receptors (NLRs), AIM2, and pyrin (27). Recognition of inflammatory ligands leads to sensor activation, oligomerization, and subsequent recruitment of adapters (28). Besides the caspase recruitment domain (CARD), the adaptor protein ASC also has a death-fold domain: the pyrin domain (PYD) (28). The structural characteristics of ASC provide support for the binding of CASP1 to the sensor, and thus the generation of CASP1 with catalytic activity, which induces pyroptosis and promotes the release of IL-1β and IL-18. It has been confirmed currently that PYD-containing NLRP3, AIM2, and pyrin recruit ASC to form inflammasomes, while CARD-containing NLRP1 and NLRC4 assemble inflammasomes *via* ASC-independent pathway, that is, directly recruiting CASP1 (27, 29, 30). Bacterial type III secretion system (T3SS) components, flagellin, abnormal or foreign DNA, dsRNA and other kinds of PAMPs and DAMPs can be specifically recognized by these sensors, and activate CASP1 (31–33). CASP1 with enzyme activity cleaves gasdermin D (GSDMD), pro-IL-1β and pro-IL-18, and promotes pyroptosis and inflammation (27, 31, 34). In addition, inflammasomes can also activate CASP8, the regulator of pyroptosis, apoptosis and necroptosis. CASP8 with enzyme activity can cleave GSDMD and GSDME (35), while CASP8 without enzyme activity can induce ASC aggregation and then activate CASP1, thus inducing pyroptosis (36).

### 2.2 Apoptosis

Apoptosis is a highly regulated process of cell death that elicits no inflammatory responses. Such stimuli as pathogen infection, DNA damage, hypoxia, and the expression of certain cancer proteins can induce apoptosis (37, 38). Apoptosis can be divided into the endogenous pathway and the exogenous pathway. The former is caused by DNA damage or endoplasmic reticulum stress, while the latter is mediated by the activation of death receptor family members (39). Apoptosis is regulated by several components, including signaling molecules, receptors, enzymes, and gene regulatory proteins, the most important of which are the BCL-2 protein family, caspase family, and tumor necrosis factor (TNF) receptor superfamily (40, 41). The Bcl-2 protein family regulates the permeability of the mitochondrial membrane, the release of substances such as cytochrome c, and the activity of caspases (42–44). CASP2/8/9 acts as an activator, while CASP3/6/7 acts as an executioner (40). As an activator of caspases, apoptotic protease-activating factor-1 (APAF-1) is closely related to endogenous apoptosis (45, 46). Cytochrome c and dATP in the cytoplasm initiate the assembly of APAF-1, and procaspase-9 is activated after binding to APAF-1 through CARD, thus activating downstream CASP3/7, and finally leading to the occurrence of apoptosis (47, 48). The TNF receptor superfamily is a key membrane receptor involved in apoptosis and an important participant in caspase-mediated apoptosis (49). Simply put, TNF receptor superfamily members recruit adaptor proteins after interacting with ligands to transmit death signals and promote apoptosis by activating CASP8 or CASP9 (49).

### 2.3 Necroptosis

Necroptosis is primarily mediated by RIPK1, RIPK3, and mixed lineage kinase domain-like protein (MLKL), which is a form of lytic cell death independent of caspases (50, 51). TNF is the main regulator of cell survival, apoptosis, and necroptosis, and the study of TNF signaling is an important pathway for recognizing necroptosis because it plays an important role in the inflammatory response induced by infection or injury (52, 53). When the activity of CASP8 is blocked, the stimulation of receptors such as TNFR1 and TLR can induce necroptosis, which involves the phosphorylation and activation of RIPK1. Some studies have shown that the catalytic activity of RIPK3 may determine whether the death pathway is apoptosis or necrotic apoptosis (53). The formation of the RIPK1-RIPK3 complex can be observed during virus infection (54). When caspase defects or inhibitors exist, the apoptosis induced by TNF and RIPK1 is transformed into necroptosis. In this process, RIPK3 regulates the phosphorylation of RIPK1 and forms a necrosome with it, and regulates the production of reactive oxygen species induced by TNF (54–56). MLKL is phosphorylated and activated by necrosome, leading to plasma membrane destruction and necroptosis (57, 58). In addition, RIPK1, RIPK3, and MLKL are also involved in the activation of inflammasomes, which is beneficial to the maturation and secretion of IL-1β and IL-18 (59, 60). In addition to TNFR, Fas, TRAILR, TLR3, and TLR4 stimulation can also induce necroptosis in the absence of CASP-8 activity (53).

### 2.4 The emergence of PANoptosis

Several decades have passed since programmed cell death (PCD) was identified. Among all the proposed forms of PCD, pyroptosis, apoptosis and necroptosis were the most well-defined in terms of the molecular machinery responsible for the initiation, transduction, and execution of cell death (61). Studies have found that there is a complex dynamic molecular network between these programs, and the processes of cell death caused by the three are not absolutely independent. Apoptosis was initially thought to be an immune-silent form, but in some cell types, it is also inflammatory (62, 63). Pyroptosis and apoptosis both involve the activation of caspase family members, indicating that the two forms may have a common evolutionary origin. As mentioned, activation of CASP1 triggers pyroptosis, a process mediated by GSDMD. Studies have found that CASP1 induces apoptosis in GSDMD-deficient cells *via* the Bid-CASP9-CASP3 axis (Bid is a member of the BCL-2 family) or other pathways (64). CASP8, as the initiator of apoptosis, can inhibit the necroptosis mediated by RIPK3 and MLKL. Notably, CASP8 scaffolding function is involved in the activation of NLRP3 inflammasome induced by dsRNA (59), while the expression of enzymatically inactive CASP8 can trigger the formation of ASC specks, the activation of CASP1, and the secretion of inflammatory cytokines (65). Both pyroptosis and necroptosis belong to the lytic cell death pathway, which means that there is likely to be a crossover between them. RIPK3 and MLKL are essential for the occurrence of necroptosis. The latest study found that in addition to the activation of MLKL, the activation of RIPK3 was also accompanied by the activation of NLRP3 inflammasome and CASP1 (66). In addition, active MLKL can also activate the NLRP3 inflammasome (67). These shreds of evidence suggest that necroptotic stimuli can trigger pyroptosis. Cell death signals are constantly evolving and diversifying to cope with pathogen infection and other risk factors. Multiple PCD pathways ensure that when particular proteins are suppressed, the body can fulfill its defensive goal *via* other means. The extensive cross-talk between pyroptosis, apoptosis, and necroptosis led to the conceptualization of “PANoptosis” (‘P’, Pyroptosis; ‘A’, Apoptosis; ‘N’, Necroptosis; and ‘optosis’, a form of programmed cell death). The phenomenon when pyroptosis, apoptosis, and necroptosis are regulated at the same time was named PANoptosis by the team of Professor Kanneganti in their studies in 2019 (11, 68, 69). PANoptosis was originally defined as “combines the main features of pyroptosis, apoptosis, and necroptosis”.

A variety of proteins can form multi-protein complexes that regulate PCD, which is based on the interaction between various protein domains that can be divided into three categories: sensing domain, assembly domain, and catalytic domain (9). For example, assembly domains include CARD, DEATH, death effector domain (DED), PYD, and receptor-interacting protein homotypic interaction motif (RHIM), among which the first four are also known as death fold domains due to their importance to the assembly of PCD-executing complexes. A variety of protein molecules, including sensors, adapters, and CASP1, are assembled into inflammasomes through PYD-PYD or CARD-CARD interactions to mediate pyroptosis (26). Apoptosis is regulated by the complex apoptosome, and a typical example is that APAF-1 senses mitochondrial-derived cytochrome C and interacts with CASP9 through the CARD domain (70). Under the inhibition of CASP8 activity, RIPK3 and RIPK1 formed a necrosome that mediated necroptosis through RHIM-RHIM interaction (71). It was concluded that the death-inducing complexes inflammasome, apoptosome, and necrosome have many core members such as NLRP3, FADD, and RIPK1 (65, 72–74). Taken together, it is not difficult to speculate that multiple proteins interacting through key domains may also be an important molecular basis for PANoptosome formation. Then in 2020, PANoptosome, a cell death-inducing complex with the molecular characteristics of pyroptosis, apoptosis, and necroptosis, was identified to induce and regulate PANoptosis (75). The PANoptosome was initially shown to contain RIPK1, ASC, NLRP3, and CASP8 (76). The subsequent study determined that RIPK3, CASP6, ZBP1, and CASP1 are also components of the PANoptosome in response to IAV infection (77). ZBP1 initiates ZBP1-PANoptosome assembly to drive inflammasome activation and cell death, and CASP6 interacts with RIPK3 to enhance the interaction between RIPK3 and ZBP1 to promote PANoptosome assembly (78). The studies of Malireddi et al. connected CASP1 (the inflammasome sensors) and CASP11 (components of pyroptosis) with CASP8, CASP7, PARP (components of apoptosis), RIPK1, and RIPK3 (components of necroptosis) (79). To sum up, these studies indicated that the PANoptosome contains molecules crucial for activating pyroptosis, apoptosis, and necroptosis to execute pro-inflammatory cell death. As a result, PANoptosis was defined as “a programmed cell death pathway induced by the PANoptosome, which provides a molecular scaffold that allows for interactions and activation of the machinery required for pyroptosis, apoptosis, and necroptosis”. However, studies focused on thoroughly elucidating the role and regulation of PANoptosis were lagging behind (79).

Recent studies highlighted the crosstalk and redundancies among pyroptosis, apoptosis, and necroptosis. The viral sensor ZBP1 regulates NLRP3 inflammasome activation and pro-inflammatory cytokine production during IAV infection through the RIPK1-RIPK3-CASP8 axis, triggering the co-occurrence of pyroptosis, apoptosis, and necroptosis (PANoptosis) in infected cells (10). Activation of CASP1, CASP8, CASP3, and phosphorylation of MLKL in macrophages infected with IAV suggest that IAV infection triggers PANoptosis in host cells. ZBP1 deletion inhibited PANoptosis, but blockade of either pyroptosis, apoptosis, or necroptosis alone failed to prevent cell death (10, 77). Studies have shown that CASP8 is a molecular converter of pyroptosis, apoptosis, and necroptosis (65). CASP8 can regulate pyroptosis through different pathways, mediating apoptosis induced by the death receptor in the exogenous pathway, and inhibiting necroptosis mediated by RIPK3 and MLKL (65, 80). In recent years, more and more molecules involved in PANoptosis have been discovered, and their roles and mechanisms are gradually clear. For example, CASP6 promotes the activation of pyroptosis, apoptosis, and necroptosis (77), and RIPK1 plays a key role in the regulation of *Yersinia*-induced PANoptosis (12). In addition to experimental evidence, the use of the STRING database enables visual analysis of the physical interactions among the molecular components of pyroptosis, apoptosis, and necroptosis, providing strong evidence for crosstalk between these three PCD pathways and also supporting the study of PANoptosis (81, 82). The results of STRING analysis indicate that CASP8 is the hub of pyroptosis, apoptosis, and necroptosis (81), which is consistent with experimental evidence showing the function of CASP8 in the three types of PCD pathways. What’s more, the role and regulation of PANoptosis in different physiological and pathological scenarios [like infections (83, 84), and cancers (85–87)] are elucidated more clearly. So, PANoptosis was described as “a unique innate immune inflammatory cell death pathway regulated by PANoptosomes, complexes that integrate molecules from other cell death pathways” (88), and the totality of biological effects in PANoptosis cannot be individually accounted for by pyroptosis, apoptosis, or necroptosis alone”.

To conclude, PANoptosis is a changing and dynamic field. As the studies go further, there is no doubt that PANoptosis will continue to get further conceptualized. It has recently been discovered that a variety of pathogens such as viruses, bacteria, fungi, and even parasites, as well as other non-infectious factors such as cytokines in tumors, may trigger PANoptosis in host cells (**Table 1**).

**Table 1:** Pathogens that can induce pyroptosis, apoptosis, and necroptosis in macrophages.

Pathogen	Pyroptosis	Apoptosis	Necroptosis	PANoptosis	References
IAV (ssRNA)	NLRP3-dependent CASP1 activation; IL-1β and IL-18 release	CASP8/3/7 activation	RIPK1/RIPK3/MLKL signaling activation	ZBP1 PANoptosome formation and activation	(10, 77)
Murine hepatitis virus (ssRNA)	NLRP3 inflammasome activation; CASP1 activation and GSDMD cleavage	CASP8/3/7 activation	phosphorylated MLKL	ZBP1 PANoptosome formation and activation	(89, 90)
SARS-CoV-2 (ssRNA)	CASP1 activation and GSDMD cleavage; IL-1β and IL-18 release	CASP8/9/3/7 activation	RIPK1/RIPK3/MLKL signaling activation	ZBP1 PANoptosome formation and activation	(89, 91, 92)
*Yersinia pestis*	CASP1 activation and GSDMD cleavage	CASP8/3/7 activation	RIPK1/RIPK3/MLKL signaling activation	RIPK1 PANoptosome formation and activation	(12)
*Staphylococcus aureus*	NLRP3 inflammasome activation; CASP1 activation and GSDMD cleavage; IL-1β and IL-18 release	CASP3 activation	RIPK1/RIPK3/MLKL signaling activation	NLRP3 inflammasome (PANoptosome) formation and activation	(93–95)
*Listeria monocytogenes*	NLRP3 inflammasome activation; CASP1 activation	CASP3 activation and Bcl-2 inhibition	RIPK1/RIPK3/MLKL signaling activation	NLRP3 inflammasome (PANoptosome) formation and activation	(96–98)
*C. albicans and A. fumigatus*	NLRP3 inflammasome activation; CASP1 activation and GSDMD cleavage	CASP8/3/7 activation	RIPK3/MLKL signaling activation	ZBP1 PANoptosome formation and activation	(11)

## 3 Molecular mechanism of PANoptosis

PANoptosis, which is closely related to pyroptosis, apoptosis, and necroptosis, shares some degree of mechanistic similarity with the three typical PCD pathways. Generally, after sensing pathogen components, sensor proteins mediate RIPK3, RIPK1, CASP8, FADD, and other proteins to assemble into a PANoptosome complex, which induces PANoptosis.

### 3.1 Evidence of PANoptosome

The concept of PANoptosome was proposed and revealed by Christian et al. in 2020, who found that molecules from pyroptotic, apoptotic, and necroptotic cell death can interact to form a single molecular complex (75). After transient overexpression of molecules known to be essential to PANoptosis in HEK293T cells, it was found that NLRP3 could co-precipitate with ZBP1, RIPK3, and RIPK1 respectively through protein interaction experiments, and CASP8, ASC, RIPK1, NLRP3, and ZBP1 could form protein complexes regardless of the presence or absence of RIPK3 (75). This method confirms the direct interaction between PANoptotic molecules, but whether these molecules can also assemble into complexes in individual cells still needs to be determined. Microscopy is a powerful and informative tool for assessing complex formations and dynamics. Wang et al. developed a method to validate and analyze PANoptosome complexes in single cells by expansion microscopy (99). This protocol allows the formation of multi-protein complexes containing ASC, CASP8, and RIPK3 to be visualized in individual cells under a variety of innate immune cell death-inducing conditions, providing evidence of PANoptosome assembly in individual cells. Up to now, different species of PANoptosomes have been identified, such as the ZBP1 PANoptosome, the AIM2 PANoptosome, and the RIPK1 PANoptosome (8, 12, 100).

### 3.2 Components of PANoptosome

The proteins that constitute PANoptosome can be divided into three types: 1) PAMPs or DAMPs sensors such as ZBP1, AIM2, and NLRP3; 2) adaptors such as ASC and FADD; 3) catalytic effectors such as RIPK1, RIPK3, CASP1 and CASP8 (9). The classification of these proteins is not absolute, for example, RIPK1 with kinase activity is required for necroptosis, where RIPK1 acts as a catalytic effector molecule; whereas the scaffold function of RIPK1 without kinase activity is required for NLRP3 inflammasome activation and cell death in TAK1-deficient cells, suggesting that RIPK1 may also act as an adaptor (76).

**Figure 2:**
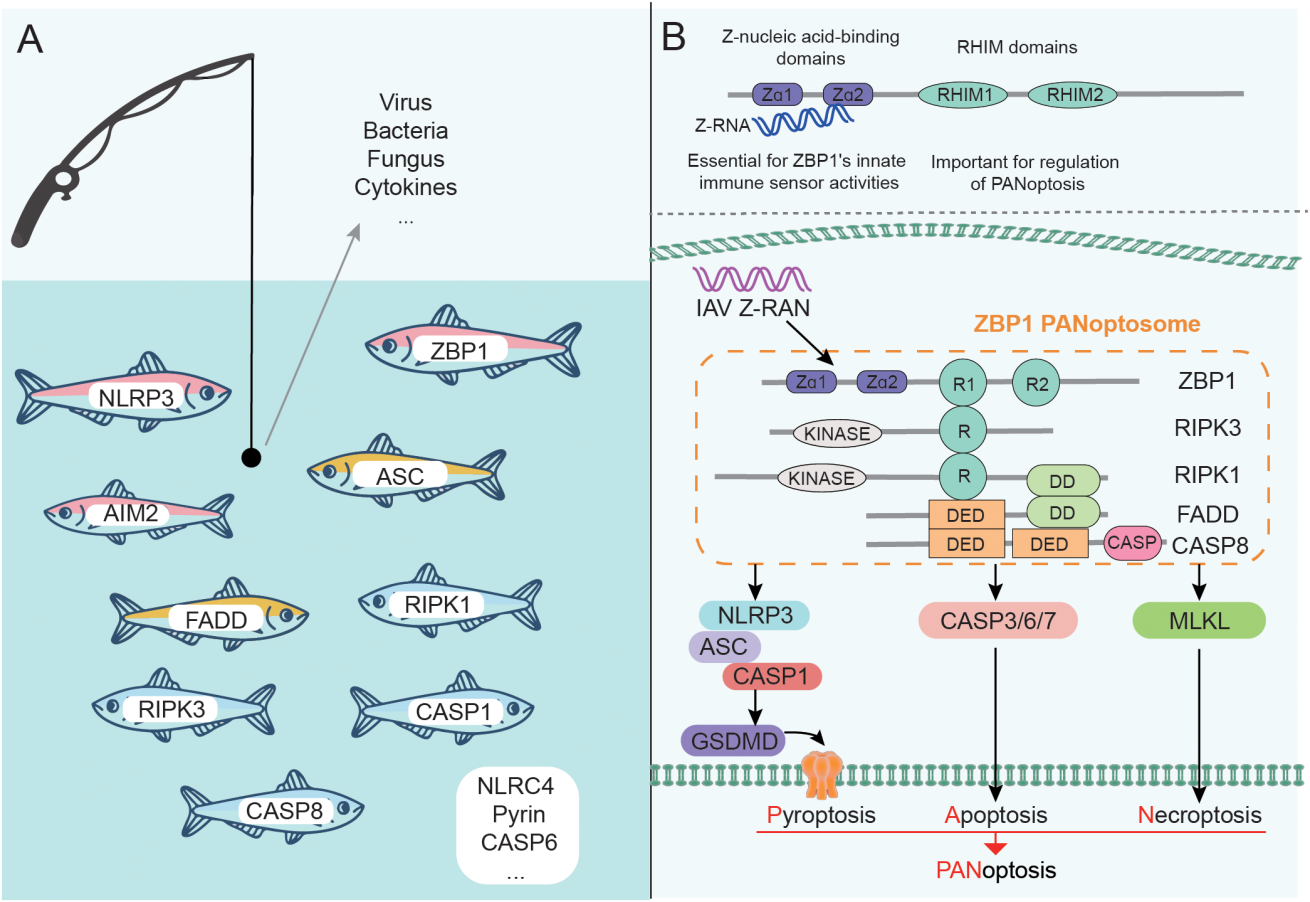
Components of PANoptosome and PANoptosis mediated by ZBP1 PANoptosome. **(A)** Many proteins can participate in the assembly of PANoptosome. The complex induced by different factors may be different, but it generally contains sensors, adapters, and effectors. **(B)** ZBP1 contains Zα and RHIM domains and is a representative sensor. A variety of proteins form ZBP1 PANoptosome through the interaction between domains, which can trigger the occurrence of PANoptosis.

With the deepening of research, it has been found that a variety of proteins participate in or coordinate the assembly of PANoptosome, and the composition of PANoptosomes induced by different factors tends to vary as well (**Figure 2A**). During *C. albicans* or *A. fumigatus* infection, ZBP1 senses special components and mediates the formation of the ZBP1/RIPK3/RIPK1/FADD/CASP8 complex (11). CASP8 and RIPK3-deficient BMDMs are largely protected from fungus-induced PANoptosis (11). TNF-α and IFN-γ produced by SARS-CoV-2 infection can promote the formation of RIPK1/RIPK3/FADD/CASP8 PANoptosome through signal transduction (91), and ZBP1 is highly likely to be involved in this process (89). Recently, it has been found that CASP6 is required for pyroptosis, apoptosis, and necroptosis but its caspase activity is not necessary during IAV infection. This is because the interaction of CASP6 with RIPK3 can promote the binding of RIPK3 and ZBP1, which in turn promotes the formation of ZBP1 PANoptosome (77). Notably, *Yersinia* induced a ZBP1-independent PANoptosome composed of RIPK1/RIPK3/CASP8, and the formation of this complex was associated with TNFR/TLR4 signaling activation and MAPKs and NF-κB signaling inhibition (12).

PANoptosis is closely related to the inflammasome, and in addition to the classical NLRP3 inflammasome, the NLRC4 inflammasome also has an important role in PANoptosis. Upon detection of the T3SS protein, NAIP binds to NLRC4 to induce assembly and activation of the NAIP/NLRC4 inflammasome, which triggers PANoptosis in infected cells *via* CASP1-GSDMD and CASP8/3/7 as well as MLKL pathways (101). The components of the PANoptosome protein complex are very complicated and interrelated. The composition of PANoptosome induced by various factors may be different, but they all can induce the co-occurrence of pyroptosis, apoptosis, and necroptosis.

### 3.3 ZBP1 PANoptosome: A representative complex that triggers PANoptosis

With further research, a variety of PANoptosomes have been identified, among which ZBP1 PANoptosome is representative (**Figure 2B**). The structural characteristics of ZBP1 are that its N-terminus contains two Z-nucleic acid binding domains (Zα domains), and the middle of the protein sequence contains two RHIM domains (100). The presence of Zα domains allows ZBP1 to act as a key innate immune sensor for Z-RNA produced by DNA and RNA viruses. Kuriakose et al. also identified ZBP1 as a sensor for nucleoprotein and polymerase subunit PB1 of IAV (10). Further studies confirmed that the C-terminal domain of ZBP1 is essential for the interaction with IAV protein, and Zα domains are also required for the interaction with PB1 (10). The structure of ZBP1 confers the ability to regulate PANoptosome formation and trigger PANoptosis in IAV infection, which has been verified: deletion of the ZBP1 Zα2 domain eliminated IAV infection-induced PANoptosis and inflammation (11, 102).

The existence of RHIM domains enables ZBP1 to interact with other RHIM-containing proteins, such as RIPK1 and RIPK3. The ZBP1-RIPK1 complex formed during IAV infection can transduce NF-κB signaling and regulate the production of IL-6 and TNF (10). The formation of the ZBP1-RIPK3 complex is conducive to recruiting RIPK1, and further forming the ZBP1 PANoptosome mainly composed of ZBP1/RIPK3/RIPK1/FADD/CASP8 (10, 77, 103). ZBP1 PANoptosome induces pyroptosis, apoptosis, and necroptosis by activating NLRP3/ASC/CASP1-GSDMD and CASP3/6/7 and MLKL pathways respectively, which ultimately triggers PANoptosis (77).

Pyroptosis, as one of the arms of PANoptosis in IAV infection, mainly relies on the NLRP3 inflammasome. Studies have found that activation of NLRP3 inflammasomes is essential in host defense against IAV and that CASP1-deficient mice have higher mortality after IAV infection (104, 105). Interestingly, inhibition of NLRP3 at late stages of infection reduced IAV infection severity (106). Therefore, the resistance to IAV depended to some extent on the activation of NLRP3 inflammasome in the early stage of infection, which was completely eliminated by the deletion of ZBP1 (10, 100, 107). It should be noted that NLRP3 inflammasome activation in other pathogen infections may not depend on ZBP1, such as the vesicular stomatitis virus (10). Previous studies have found that RIPK1 and RIPK3 are required for NLRP3 inflammasome activation induced by RNA viruses, including IAV (108). Mechanistically, infection triggers the formation of the RIPK1-RIPK3 complex, which promotes phosphorylation of dynamin-related protein 1 (DRP1) and translocation to mitochondria, thereby driving mitochondrial damage and NLRP3 inflammasome activation (108). The formation of the ZBP1-RIPK3 complex is conducive to the recruitment of RIPK1 and other proteins. Therefore, the formation of ZBP1 PANoptosome during IAV infection is very important for the activation of NLRP3 inflammasome. CASP8, which is involved in constituting ZBP1 PANoptosome, also has a regulatory effect on NLRP3 inflammasome activation. Compared with the deletion of RIPK3, the deletion of RIPK3 and CASP8 is more unfavorable to inflammasome activation, consistent with the effect of ZBP1 loss during IAV infection (10). Structurally, CASP8 has no domain that can interact with ZBP1, so it needs to rely on RIPK1 for recruitment through FAS-associated protein with dead domains (FADD). GSDMD cleavage was impaired in IAV-infected *Ripk3*^-/-^and *Ripk3*^-/-^*Casp8*^-/-^BMDMs compared to WT BMDMs, suggesting that RIPK3 and CASP8 are the main components of ZBP1 PANoptosome that mediate pyroptosis (77). MLKL is a substrate of RIPK3 and simultaneously an executor of necroptosis. However, experiments have shown that the deaths of WT and *Mlkl*^-/-^ BMDMs infected with IAV are almost the same (10), indicating that MLKL is not necessary for cell death caused by ZBP1 and RIPK3-mediated IAV infection, which provides evidence for the existence of other forms of cell death. Compared with the wild type, the activation of CASP8/3/7 in *Zbp1^-/-^* BMDMs infected with IAV was abrogated, confirming that ZBP1 indeed mediates apoptosis in IAV infection (10).

It was found that fibroblasts and BMDMs lacking RIPK3 did not die due to IAV infection (10). This strongly demonstrates the importance of RIPK3 in ZBP1 PANoptosome, which plays a dominant role in the activation of the NLRP3 inflammasome, the phosphorylation of MLKL, and the activation of CASP8/3/7. Furthermore, it was confirmed that concurrent use of CASP8 inhibitors and MLKL inhibitors prevented pyroptosis, apoptosis, and necroptosis of WT BMDMs caused by IAV infection (109). We hypothesized that this may be due to the involvement of CASP8 in the regulation of both pyroptosis and apoptosis, together with the inhibition of MLKL preventing necroptosis from occurring. In short, ZBP1, RIPK3, RIPK1, FADD, and CASP8 constitute ZBP1 PANoptosome, which together coordinate pyroptosis, apoptosis, and necroptosis with parallel contribution as well as inflammatory response in IAV infection, and infected cells do not die unless three pathways are blocked at the same time.

### 3.4 AIM2 PANoptosome: A complex containing multiple sensors

It is known that AIM2 can detect dsDNA released during the pathological attack and cellular perturbation, and its recognition of dsDNA leads to the assembly of AIM2 inflammasome and the occurrence of pyroptosis, which plays an important role in infectious diseases, inflammatory diseases, and cancer (110, 111). Herpes simplex virus 1(HSV1) and *Francisella novicida* are known pathogens that can activate the AIM2 inflammasome (8, 112). Recently, it has been found that the function of AIM2 is not limited to activating inflammasome and triggering pyroptosis. For example, AIM2 and NLRP3 can form a dual cytoplasmic surveillance system, which has a stronger protective effect on *A. fumigatus* infection than either of the two alone (113).

The role of AIM2 in PANoptosis has been identified as regulating pyrin and ZBP1 to drive PANoptosis and inflammatory responses in HSV1 or *F. novicida* infection (8). It was found that the expressions of pyrin and ZBP1 in *Aim2*^–/–^, *Asc*^–/–^, *Casp1*^–/–^ BMDMs infected with HSV1 or F. novicida were decreased, but not in *im2*^–/–^, *Asc*^–/–^, *Casp1*^–/–^BMDMs infected with IAV (8). Interferon (IFN)-β production was noted in HSV1 or *F. novicida* infected WT BMDMS (8), and IFN signaling has previously been found to promote the expression of pyrin and ZBP1 (10, 75). IFN-β was not found in *Aim2*^–/–^, *Asc*^–/–^, *Casp1*^–/–^BMDMs, and the expression of pyrin and ZBP1 recovered after IFN-β supplementation (8), which suggested that the production of IFN-β mediated by AIM2/ASC/CASP1 pathway is necessary for the expression of pyrin and ZBP1 in HSV1 or *F. novicida* infection. Infection with HSV1 or *F. novicida* can induce AIM2-dependent and NLRP3/NLRC4-independent activation of CASP1, followed by the release of IL-1β and IL-18 and cell death (8). However, under the same condition, the inflammasome activation and cell death were decreased in *Mefv*^–/–^ BMDMs, suggesting that the function of AIM2 is driven by pyrin to some extent (8). In addition, the absence of ZBP1 also attenuated the effect of AIM2. Compared with WT BMDMs, the activation of CASP1/GSDMD/GSDME, CASP8/3/7, and RIPK3/MLKL in *Mefv*^–/–^ and *Zbp1*^–/–^ BMDMs decreased after infection with HSV1 or *F. novicida*, and completely inactive in *Aim2*^–/–^ and *Mefv*^–/–^*Zbp1*^–/–^ BMDMs (8). These results suggested that ZBP1 and pyrin synergistically drive AIM2-mediated PANoptosis in HSV1 or *F. novicida* infection. HSV1 or *F. novicida* infection activates AIM2 and promotes the formation of PANoptosome consisting of AIM2, pyrin, and ZBP1 as well as molecules such as ASC, RIPK3, RIPK1, FADD, CASP1, and CASP8, which is necessary for cell death (PANoptosis). The limited assembly of AIM2 PANoptosome, such as the lack of AIM2, pyrin, or ZBP1, will lead to the death of infected mice (8).

Notably, HSV1 and *F. novicida*, while activating AIM2, also attract the ZBP1 Zα domain and inhibit Rho-GTP activity, thus enabling the assembly of PANoptosome containing a variety of sensors (8). Similarly, other pathogens that can bind to multiple sensors may also form PANoptosomes with shared central components.

## 4 The regulation of PANoptosis

**Figure 3:**
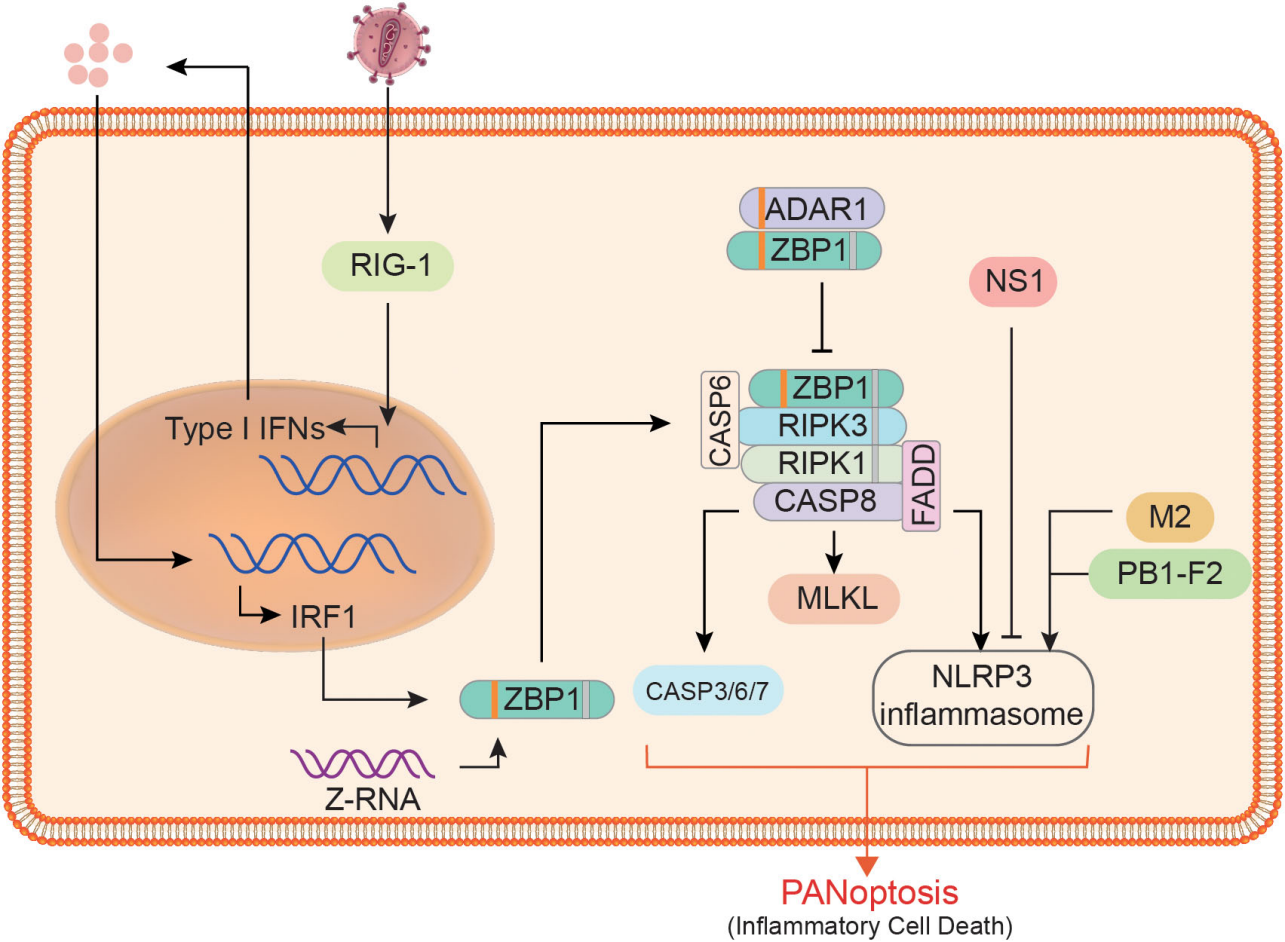
Regulation of ZBP1 PANoptosome formation and downstream effector activity. At the expression level, ZBP1 is affected by RIG1, type I IFN, and IRF1. At the assembly level, ADAR1 limits the interaction between ZBP1 and RIPK3, whereas CASP6 does the opposite. The downstream of ZBP1 PANoptosome, the activities of pyroptosis, apoptosis, and necroptosis executors are affected by many factors. For example, non-structural protein 1 (NS1), matrix protein 2 (M2), and PB1-F2 can affect the activation of the NLRP3 inflammasome.

PANoptosis, like the three related canonical PCD pathways (pyroptosis, apoptosis, and necroptosis), must also be a programmed and regulated cell death pathway, which is one of its important characteristics. Cell death is essential for host defense, but it must be controlled or it is likely to cause more damage to the organism. TNF-α and IFN-γ produced during SARS-CoV-2 infection were found to induce PANoptosis, causing lethal cytokine shock, while PANoptosis inhibition was protective in infected mice (114). Therefore, regulating the PANoptosis process to control proper cell death is particularly important for the resistance to risk factors and the protection of body health. We suggest that the regulation of PANoptosis mainly involves the recognition of DAMPs or PAMPs, the preparation and assembly of PANoptosome components, and the regulation of various effector activities. Here we focus on the regulation of ZBP1 PANoptosome formation (**Figure 3**).

IRF1 is recognized as a regulator of cell death, and it also plays a key regulatory role in the process of cell death through the PANoptosis pathway (88, 115). As a transcriptional regulator of ZBP1, IRF1 undoubtedly acts as an upstream regulator of PANoptosis during infection with pathogens such as IAV (116). When IRF1 is deficient, the expression of ZBP1 in infected cells and the activation of downstream proteins, including CASP1/3/8 and MLKL, will decrease significantly (116). Impaired pyroptosis, apoptosis, and necroptosis occurred in the colon of *Irf1*^–/–^mice, which resulted in reduced cell death (117). During IAV infection, IRF1 expression is largely dependent on type I IFN, indicating that the synthesis of ZBP1 and even other sensors are also regulated by IFN signaling (8, 10, 75). ZBP1 expression was not detected in cells without the INF receptor (INFAR1/2) or its downstream signaling factors (such as IRF9 and STAT1) (10, 107). RIG1 may be a type I IFN sensor in BMDMs, and its deletion will reduce ZBP1 expression in IAV infection to some extent (107). Since RIG-1 is dispensable for ZBP1 expression, additional sensors may exist that require further validation. In addition to the regulation of ZBP1 expression, its posttranslational modification is also a key factor affecting PANoptosis. Studies have found that ZBP1 ubiquitination significantly increases after IAV infection (107), indicating that ubiquitination modification of ZBP1 can regulate the assembly of ZBP1 PANoptosome. Furthermore, no ubiquitination modification of ZBP1 was observed following IFN- treatment. Aside from the preparations for regulating the PANoptosome components, there are a number of factors that do not affect their synthesis but can control their assembly. For example, adenosine deaminase acting on RNA 1 (ADAR1) can restrict ZBP1-mediated PANoptosis and promotes tumorigenesis (85). P150 and p110 are two isoforms of ADAR1; the former contains a Zα domain and is an IFN-inducible form. It was found that ADAR1-p150 in the cytoplasm could compete with RIPK3 to bind ZBP1, thus limiting cell death and that ZBP1 can function normally when there is no ADAR1-p150 in the cytoplasm (85). The assembly of PANoptosome is also regulated by many substances. For example, CASP6 mentioned above can interact with RIPK3, a key component in the complex (77), where CASP6 may provide a scaffold for the assembly.

In addition to host factors, pathogen components also play a significant role in PANoptosis. For example, the ion channel activity of matrix protein 2 of the influenza virus enables the export of hydrogen ions from the Golgi apparatus, which is the trigger for the formation of the NLRP3 inflammasome (118). Further to that, PB1-F2 and non-structural protein 1 of the influenza virus have been identified as NLRP3 inflammasome regulators (119, 120). In conclusion, PANoptosis, a programmed cell death process, is regulated by a variety of factors, which also provides a basis for the artificial regulation of PANoptosis for the treatment of various diseases.

## 5 PANoptosis and diseases

PANoptosis has become a research hotspot in many diseases, such as pathogen infection, immune diseases, and even cancer. Pyroptosis is related to the occurrence and development of various diseases caused by a variety of pathogens (121). Pyroptosis is also well-studied in tumors, and it has been proposed that pyroptosis can not only inhibit tumor cell proliferation but also form a suitable microenvironment for tumor cell growth and promote tumor growth (122). Apoptosis plays a key role in the progression of several neurological diseases such as Alzheimer’s disease, Parkinson’s disease, and Huntington’s disease, while apoptosis is also considered an anticancer mechanism (123, 124). Similarly, necroptosis has a further role in promoting cell death and neuroinflammation in a variety of neurodegenerative diseases (50). The role of necroptosis in cancer is also extremely complex, some cancer cells survive by escaping necroptosis, and in some cases, it can also promote tumorigenesis and cancer metastasis (53). The relationship between PCD and disease development and treatment cannot be generalized, and this complicated relationship also provides a theoretical basis for the targeted regulation of PCD in the treatment of various diseases. As an advanced version of the three PCD pathways, PANoptosis undoubtedly plays a greater role in the treatment of diseases.

Understanding the mechanisms and regulation of PANoptosis is fundamental to the treatment of related diseases, and so far there is evidence of a relationship between PANoptosis regulation and the development or progression of certain diseases.

On the one hand, the occurrence of PANoptosis contributes to inhibiting the development of certain diseases, especially cancer. Adar1^fl/fl^LysM^cre^ mice were found to be resistant to the development of colorectal cancer and melanoma, Adar1^fl/fl^LysM^cre^ mice were found to be resistant to the development of colorectal cancer and melanoma due to the pro-tumorigenic effect of ADAR1 by inhibiting PANoptosis through binding to the Zα2 domain of ZBP1 (85). Cysteine desulfurase (NFS1) deficiency significantly enhanced the sensitivity of colorectal cancer cells to oxaliplatin due to the synergistic effect of NFS1 deficiency with oxaliplatin treatment, triggering PANoptosis by increasing intracellular levels of reactive oxygen species (125). Although the exact mechanism is unclear, certainly, all three PCD pathway inducers are significantly increased in cancer cells during oxaliplatin treatment after *NFS1* knockdown (125). Pan et al. identified three distinct patterns of PANoptosis in gastric cancer patients with different molecular and clinical characteristics (87). The construction and use of the PANoptosis scoring system allow for the effective prediction of patient immunotherapy response and prognosis (87). PANoptosis plays a role in a variety of cancers, and we believe that by increasing the expression and activity of key components of the PANoptosome and reducing inhibitors, we can effectively induce cancer cell death and improve the therapeutic efficacy of chemotherapy.

On the other hand, excessive cell death due to PANoptosis is responsible for the development of the disease. The study demonstrates that interferon induces ZBP1-mediated PANoptosis in human and mouse macrophages during SARS-CoV-2 infection (89). And increased expression of ZBP1 in immune cells of patients who succumbed to SARS-CoV-2 infection compared to those who recovered, suggesting an important link between PANoptosis and disease progression (89). In acute lung injury/respiratory distress syndrome, alveolar epithelial damage is an important determinant of disease severity. It was found that miR-29a-3p agomir injection could down-regulate ZBP1, GSDMD, CASP3, CASP8, and MLKL, reduce the level of inflammatory factors in the lung and inhibit the PANoptosis of alveolar epithelial, thereby alleviating lung injury (126). In addition, through data integration and analysis, Xiong et al. discovered that PANoptosis is present in ischemic brain injury and can be used as a therapeutic target for various central nervous system diseases (69).

The relationship between cell damage or death and the development of specific diseases is not difficult to imagine, but how to regulate this process is a major issue. In the case of PANoptosis, what we need to do is to regulate the expression and activity of key components of the PANoptosome, increasing or decreasing the inhibitors, which is the direction and key to using PANoptosis regulation for disease treatment. What’s more, the characterization of PANoptosis should not be overlooked, and the establishment of a disease-specific PANoptosis scoring system can assist in selecting treatment strategies and determining prognosis.

## 6 Summary and prospect

PANoptosis, in essence, is not only pyroptosis, apoptosis, and necroptosis occurring together in cells, but also a balanced and complementary cell death pathway. When some pathways are inhibited, other signals may be activated to achieve the same effect or enhance other death pathways. Previous studies have confirmed that the absence of NLRP3 inflammasome leads to increased apoptosis and necroptosis (90). The composition and assembly of PANoptosome are extremely flexible, which ensures that core components of different cell death pathways can be recruited to perform regulated inflammatory cell death. Since the concept of PANoptosis was introduced, no study has clearly stated whether this inflammatory cell death occurs in the same cell or in a population of cells, which may be difficult to prove but is undoubtedly an inevitable problem. We believe that PANoptosis should occur in the same cell. First, returning to the concept itself, PANoptosis cannot be replaced by one or two of pyroptosis, apoptosis, and necroptosis. Secondly, the formation and activation of PANoptosome in a single cell provides support for PANoptosis, a form of inflammatory cell death driven by PANoptosome, to occur in the same cell. Finally, the same cells in the same environment should die similarly.

A variety of sensors in the human body provide a guarantee for the perception of DAMPs, PAMPs, and other risk factors, which is also an important link to triggering PANoptosis. A series of signal-mediated production of proteins required for the assembly of PANoptosome is the guarantee for the smooth progress of PANoptosis. PANoptosis, an intricate procedural process, is inevitably affected by host factors and external factors such as pathogen proteins, which mainly affect the synthesis of related proteins and complex assembly, as well as the activity of various effectors. From sensing risk factors to triggering cell death and generating multiple effects, many proteins and signals are involved; therefore, modulation of the expression of these proteins and signaling pathways is a feasible way to regulate PANoptosis. Pyroptosis, apoptosis, and necroptosis do have therapeutic potential for a variety of diseases, such as infectious diseases, cancer, and neurological diseases, but are often double-edged swords. We suggest that PANoptosis is also highly likely to have multiple roles in multiple diseases, and therefore targeting to promote or inhibit this process according to micro-environmental changes is expected to be a highly effective therapeutic strategy. More importantly, compared with the regulation of pyroptosis, apoptosis, and necroptosis alone, the strategies and methods for regulating PANoptosis may be more convenient, and the results may be better.

Although PANoptosis has promising prospects for the treatment of multiple diseases, there are still many problems to be solved. The mechanism of PANoptosis is not clear enough, and the function of the proteins constituting PANoptosome remains to be further studied. The therapeutic response of PANoptosis and other treatments such as radiotherapy, chemotherapy, and the relationship of PANoptosis to specific immunotherapy is not fully understood. For the regulation of PANoptosis, the selection of inhibitors or inducers, the specificity and effectiveness of regulation, and the extent of the regulation still need to be explored. In a nutshell, as a highly correlated form of programmed cell death, PANoptosis plays a critical role in many diseases such as infection, cancer, neurodegenerative diseases, inflammatory diseases, and so on. Further research into the molecular mechanisms and regulatory mechanisms of this process will have new implications for the treatment of a variety of diseases.

## Author contributions

PZ and Z-RK wrote the original draft. PZ, Z-RK and J-XC participated in writing and editing the review. PZ and S-JL prepared the figures. X-LF and T-LM edited the manuscript. All authors contributed to the article and approved the submitted version.

## Glossary

**Table:**

AIM2	absent in melanoma 2
ADAR1	adenosine deaminase acting on RNA 1
APAF-1	apoptotic protease-activating factor-1
ASC	apoptosis-associated speck-like protein containing a caspase recruitment domain
BMDMs	bone marrow-derived macrophages
CARD	caspase recruitment domain
CASP1	caspase-1
DED	death effector domain
FADD	Fas-associated protein with death domain
GSDMD	gasdermin D
HSV1	Herpes simplex virus 1
IAV	influenza A virus
IBD	inflammatory bowel disease
IRF1	Interferon regulatory factor 1
IFN	Interferon
MLKL	mixed lineage kinase domain-like protein
NLRs	nucleotide-binding domain-like receptors
NLRP3	NOD-like receptor family pyrin domain containing 3
PCD	programmed cell death
PYD	pyrin domain
RHIM	receptor-interacting protein homotypic interaction motif
RIPK3	receptor-interacting protein kinase 3
TAK1	TGF-β activated kinase-1
TNF	tumor necrosis factor
T3SS	type III secretion system
Zα domains	Z-nucleic acid binding domains
ZBP1	Z-DNA-binding protein 1
